# Optimal biological dose: a systematic review in cancer phase I clinical trials

**DOI:** 10.1186/s12885-021-07782-z

**Published:** 2021-01-13

**Authors:** J. Fraisse, D. Dinart, D. Tosi, C. Bellera, C. Mollevi

**Affiliations:** 1 Unité de Biométrie, Institut du Cancer Montpellier (ICM), Université de Montpellier, 208 rue des Apothicaire, 34298 Montpellier Cedex 5, France; 2 Inserm CIC1401, Module Epidémiologie clinique, Institut Bergonié, Bordeaux, France; 3 Institut Desbrest d’Epidémiologie et de Santé Publique, UMR Inserm - Université de Montpellier, Montpellier, France

**Keywords:** Phase 1 clinical trial, Cancer, Dose finding study, Optimal biological dose

## Abstract

**Background:**

Classical phase 1 dose-finding designs based on a single toxicity endpoint to assess the maximum tolerated dose were initially developed in the context of cytotoxic drugs. With the emergence of molecular targeted agents and immunotherapies, the concept of optimal biological dose (OBD) was subsequently introduced to account for efficacy in addition to toxicity. The objective was therefore to provide an overview of published phase 1 cancer clinical trials relying on the concept of OBD.

**Methods:**

We performed a systematic review through a computerized search of the MEDLINE database to identify early phase cancer clinical trials that relied on OBD. Relevant publications were selected based on a two-step process by two independent readers. Relevant information (phase, type of therapeutic agents, objectives, endpoints and dose-finding design) were collected.

**Results:**

We retrieved 37 articles. OBD was clearly mentioned as a trial objective (primary or secondary) for 22 articles and was traditionally defined as the smallest dose maximizing an efficacy criterion such as biological target: biological response, immune cells count for immunotherapies, or biological cell count for targeted therapies. Most trials considered a binary toxicity endpoint defined in terms of the proportion of patients who experienced a dose-limiting toxicity. Only two articles relied on an adaptive dose escalation design.

**Conclusions:**

In practice, OBD should be a primary objective for the assessment of the recommended phase 2 dose (RP2D) for a targeted therapy or immunotherapy phase I cancer trial. Dose escalation designs have to be adapted accordingly to account for both efficacy and toxicity.

**Supplementary Information:**

The online version contains supplementary material available at 10.1186/s12885-021-07782-z.

## Background

The primary objective of phase 1 cancer clinical trials is to assess the maximum tolerated dose (MTD) based on the dose limiting toxicity (DLT) evaluated in most of the cases on the first cycle of treatment, the safety profile and the recommended phase 2 dose (RP2D) [1]. Most dose-finding designs available for phase 1 cancer clinical trials were initially developed in the context of cytotoxic conventional agents. These methods are based on an underlying hypothesis which implies that the dose of a cytotoxic drug is related to the toxic response via an increasing monotonic relationship [2]. With the emergence of molecular targeted agents and immunotherapies and given their specific mechanism of action, this paradigm has been modified. Severe toxicities are rare, often delayed in subsequent treatment cycles, preventing the MTD from being reached [3]. As such, dose-finding designs based only on a toxicity endpoint may not be appropriate anymore. In this context, the concept of optimal biological dose (OBD) has been introduced, which accounts for efficacy in addition to toxicity. Assessing the OBD instead of the classical MTD thus appears particularly relevant for modern phase I trials [4].

OBD is generally defined as the lowest dose providing the highest rate of efficacy while being safely administered. To our knowledge, there is however no consensus on the efficacy endpoint to be accounted for in the OBD, nor on the most appropriate dose escalation strategy to apply when assessing OBD.

Several efficacy endpoints and dose escalation designs have been proposed in the context of OBD, as it requires to simultaneously account for efficacy and toxicity. With regards to dose-escalation designs, Piantadosi and Liu proposed a variant of the continual reassessment method (CRM) dose-escalation design [5], which models the dose-efficacy curve via an auxiliary pharmacokinetics (PK) measurement (area under the curve, AUC) using a two-parameter logistic dose-efficacy model [6]. Braun proposed also to extend the CRM to a bivariate trial design for two competing outcomes: toxicity and disease progression [7]. Bekele and Shen proposed a bayesian approach to jointly model the dose-toxicity and dose-efficacy curves [8]. The authors expressed toxicity using a binary variable (presence or absence of toxicity), while efficacy was modeled using a continuous biomarker expressing the concentration of a target protein. This sequential method specifically models the correlation between toxicity and efficacy via a latent Gaussian variable. Dragalin and Fedorov proposed a similar method where patient response is characterized by two dependent binary outcomes, one for efficacy and one for toxicity, using either a bivariate logistic model or a Cox bivariate binary model [9, 10]. Houede et al. proposed an outcome-adaptive bayesian design with toxicity and efficacy characterized by ordinal variables, with efficacy defined as complete response, partial response, stable disease or progressive disease, and toxicity defined as a three-level ordinal variable representing the worst severity of adverse events [11]. Individual probabilities of severe toxicity and tumor response are then sequentially jointly re-estimated.

Overall, these developments highlight the heterogeneity in terms of both efficacy endpoints and dose escalation designs in the context of OBD. Efficacy may rely on pharmacokinetic or pharmacodynamic (PD) endpoints, clinical or radiological measures, or biomarkers such as immune response. Similarly, methodological developments have led to various phase I designs (bivariate models vs joint models, binary vs ordinal variables, etc.). The development of novel therapeutic anti-cancer agents has challenged traditional approaches conducting phase 1 trials. The objective of the present work was therefore to provide an overview of recent phase 1 cancer clinical trials relying on the concept of OBD, with a particular focus on (i) how efficacy is accounted for in the definition of OBD, and (ii) dose-escalation designs allowing for the estimation of OBD.

## Methods

### Selection

The systematic review involved two steps: selection of relevant manuscripts and data extraction. We performed a systematic review through a computerized search of the MEDLINE database to identify cancer early phase clinical trials that relied on OBD. The search algorithm was the following: (((((“Optimal” AND (“Biologic” OR “Biological”) AND “Dose”) AND “cancer”[Filter]) AND “humans”[Filter]) AND (“2000/01/01”[Date - MeSH]: “2019/12/31”[Date - MeSH]))) AND “clinical trial”[Filter]. We selected relevant publications based on a two-step process using a standardized data extraction grid designed and validated by two readers who independently checked both steps of the selection process. Discrepancies were resolved by mutual consensus. In the first step, general information was retrieved based on the abstract. Publications were ineligible if the abstract presented at least one of the following characteristics: letter/comment to the editor; conference abstract; not conducted in humans; not related to cancer; not a phase I trial. In the second step of the selection process, we read the full manuscripts of the selected abstracts. Publications were ineligible if they included at least one of the following characteristics: absence of an efficacy endpoint, absence of a toxicity endpoint; methodological paper. Results of the selection process are presented following the PRISMA guidelines for the reporting of systematic reviews and meta-analyses [12, 13].

### Data collection and analysis

For full manuscripts that met eligibility criteria, we collected information regarding general characteristics of the articles: title of the article, phase of the study, localization of the cancer, molecules tested (single or association), type of therapeutic agents, principal and secondary objectives, primary and secondary endpoints (among toxicity and efficacy) and dose-finding design. We collected definitions for the OBD, MTD, DLT, as well as observation period for assessment of the DLT.

We provide a descriptive analysis of the publications. Quantitative variables were reported using descriptive statistics (median, minimum and maximum). For qualitative variables, we provided counts (N) and proportions (%) of each modality. Analyses were performed using the STATA software (version 16; STATA, College Station, TX).

## Results

### Trial selection

**Fig. 1:**
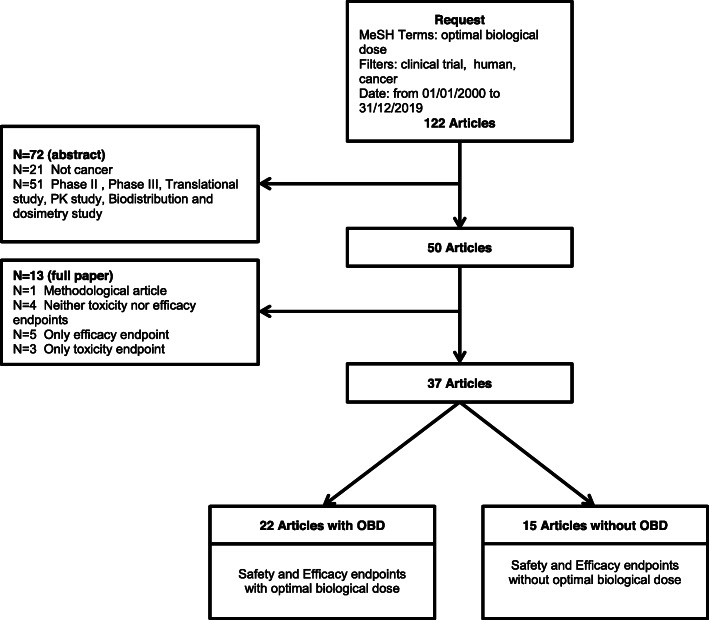
Study selection

The algorithm initially retrieved a total of 122 publications (Fig. 1). We excluded 72 manuscripts following the first step of the selection process, leading to 50 manuscripts (21 publications did not report on cancer; 51 publications did not report on a phase 1 trial). Following the second step of the review process, we subsequently excluded one manuscript specifically focusing on methodological issues, and 12 manuscripts due to absence of either an efficacy or toxicity endpoint or both. Based on the remaining 37 articles, 22 referred to the OBD and 15 did not refer to the OBD. We provide below a description of these two subgroups of manuscripts.

### Characteristics of the trials

Characteristics of the selected trials are described in Table 1. The 37 trials were either phase I (78.4%) or phase I/II trials (21.6%). The 22 manuscripts reporting on OBD were more frequently phase I trials (*n* = 16; 72.7%) as well as the 15 manuscripts that did not report on OBD (*n* = 13; 86.7%). Most trials reported on solid tumors (*n* = 28, 75.7%), half of which were related to multiple organs (*n* = 14/28, 50.0%). The cancer site did not vary substantially between trials reporting on OBD and those that did not. In articles reporting on OBD, about half reported on a single molecule and half on a combination of therapies. On the other hand, most of the articles that did not report on OBD focused more often on a single molecule (80.0%). In articles reporting on OBD, the primary objective was either the MTD (31.8%), the OBD (50.0%) or both (18.2%). Primary endpoint was the assessment of DLT (31.8%), or a combination of endpoints involving DLT assessment and either PK, biological or clinical response (59.0%). In articles that did not report on OBD, MTD was the primary objective for all manuscripts, and primary endpoint was DLT assessment. More than 80% of the articles mentioned a dose escalation design based on an algorithmic method (3 + 3, sequential cohorts or modified Fibonacci) associated with an observation period for DLT during the first cycle of treatment.

**Table 1:** Characteristics of studies included in review

	Article with OBD		Article without OBD		Total	
	***N*** = 22	%	***N*** = 15	%	***N*** = 37	%
**Trial**						
Phase I	16	72.7	13	86.7	29	78.4
Phase I/II	6	27.3	2	13.3	8	21.6
**Location**						
Hematologic cancer	2	9.1	2	13.3	4	10.8
Solid tumor	17	77.3	11	73.3	28	75.7
Melanoma	3	13.6	0		3	8.1
Solid tumor + Melanoma	0		1	6.7	1	2.7
Solid tumor + Hematologic cancer	0		1	6.7	1	2.7
**Therapeutic schedule**						
Molecule	11	50.0	12	80.0	23	62.2
Association of molecules	11	50.0	3	20.0	14	37.8
**Principal objective**						
MTD	7	31.8	15	100	22	59.5
OBD	11	50.0	0		11	29.7
OBD/MTD	4	18.2	0		4	10.8
**Primary endpoint**						
DLT	7	31.8	15	100	22	59.5
DLT + (PK + clinical response)	1	4.5	0		1	2.7
DLT + biological target	8	36.4	0		8	21.6
DLT + clinical response	3	13.6	0		3	8.1
Biological target	2	9.1	0		2	5.4
Toxicity + biological target	1	4.5	0		1	2.7
**Observation period**						
Not defined	5	25.0	2	13.3	7	20.0
First cycle	11	55.0	13	86.7	24	68.6
Other	4	20.0	0		4	11.4
Missing	2		0		2	
**Dose-escalation method**						
3 + 3	6	27.3	7	46.7	13	35.1
Modified fibonacci	2	9.1	3	20.0	5	13.5
CRM	2	9.1	0		2	5.4
Consecutive / Sequential cohorts	9	40.9	3	20.0	12	32.4
Other	3	13.6	2	13.3	5	13.5
**DRP2**						
No	16	72.7	10	66.7	26	70.3
Yes	6	27.3	5	33.3	11	29.7
**Secondary objective**						
OBD	7	31.8	0		7	18.9
Other	15	68.2	15	100	30	81.1
**Secondary endpoint associated with OBD**						
DLT + biological target	1	14.3	0		1	14.3
DLT + clinical response	4	57.1	0		4	57.1
Toxicity + biological target	2	28.6	0		2	28.6
**Secondary endpoint**: **PK/PD**						
No	10	45.5	3	20.0	13	35.1
PK	4	18.2	5	33.3	9	24.3
PD	0		1	6.7	1	2.7
PK/PD	8	36.4	6	40.0	14	37.8
**Secondary endpoint: Clinical response**						
No	6	27.3	1	6.7	7	18.9
Yes	16	72.7	14	93.3	30	81.1
**Secondary endpoint: Immune response**						
No	18	81.8	13	86.7	31	83.8
Yes	4	18.2	2	13.3	6	16.2
**Secondary endpoint: Survival outcome**						
No	13	59.1	10	66.7	23	62.2
Yes	9	40.9	5	33.3	14	37.8

### Endpoints and methods considered in trials reporting on OBD

The detailed characteristics of the 22 trials reporting the OBD are described in Table 2. Trials focused mainly on targeted therapies (*n* = 12/22; 54.5%) and immunotherapies (*n* = 4/22; 18.2%). OBD was traditionally defined as the smallest dose maximizing an efficacy criterion such as biological activity. Efficacy was usually defined based on a biological target when OBD was the primary objective (*n* = 11 out of 15, 73.3%) as well as secondary objective (*n* = 3 out of 7, 42.9%). Biological endpoints included biological response (e.g. variation of biomarkers such as cells, proteins, microvessel density), immune cells count (cytokines, lymphocytes) for immunotherapies, or biological cell count (blood, urine) for targeted therapies. Few articles combining immunotherapy with biological agents and clearly mentioning the OBD have been identified (*n* = 3). For those particular cases, OBD was assessed using conventional DLT for safety and IR for efficacy. IR was specifically defined according to the studied molecule. The objective was to find among the safest doses the one with the highest immunogenicity [16, 36].

**Table 2:** Detailed characteristics of studies with OBD

Article	Location	Type of treatment	Studied molecules	Escaladed molecules	Phase	Principal objective	Primary endpoint	Statistical design	Secondary endpoints				
									OBD^**a**^	Clinical response	Immune response	Survival outcome	PK and/or PD
[14]	Solid	Immunotherapy	Interleukin 2 13-cis Retinoic Acid	Interleukin 2	Phase I	OBD	DLT + IR	Consecutive/sequential cohorts		CR (WHO response criteria)	IR (changes of total T- and T-helper cell counts)	TTP, OS	
[15]	Solid	Immunotherapy	Interleukin 12 Trastuzumab	Interleukin 12	Phase I	MTD	DLT	3 + 3	DLT + IR	CR (not specified)	IR (induction of systemic NK cell-derived cytokines)		
[16]	Melanoma	Immunotherapy	Set of combination immunotherapies	Set of combination immunotherapies	Phase I/II	OBD	DLT + IR	CRM for two binary endpoints			IR (levels of peptide-reactive CD8+ cells)		
[17]	Melanoma	Immunotherapy	Recombinant human IL-12 Melan-A and influenza peptides	Recombinant human IL-12	Phase I	OBD	Toxicity + IR	Other		CR	IR (cytotoxic lymphocyte response + cutaneous responses)		
[18]	Solid	Metabolic therapy	Pegylated recombinant human arginase 1	Pegylated recombinant human arginase 1	Phase I	OBD	DLT + plasma arginine depletion	Modified fibonacci		CR (RECIST criteria)		PFS, OS	PK/PD
[19]	Melanoma	Metabolic therapy	High dose paracetamol Carmustine	High dose paracetamol Carmustine	Phase I	MTD	DLT	3 + 3	Toxicity + effects on GSH levels	CR			PK
[20]	Solid	Oncolytic Virus therapy	NV1020	NV1020	Phase I/II	MTD	DLT	Consecutive/sequential cohorts	DLT + CR	CR (RECIST criteria)		TTP, OS	PK
[21]	Solid	Radiotherapy	Carbon ion	Carbon ion	Phase I/II	OBD	DLT + local control rate	Other				OS, cause specific survival	
[22]	Solid	Radiotherapy	Carbon ion	Carbon ion	Phase I	MTD	DLT	3 + 3	DLT + CR	CR (RECIST criteria)		PFS, OS	
[23]	Solid	Radiotherapy	Carbon ion	Carbon ion	Phase I	OBD	DLT + tumor response at 6 months	Other		Local control		OS	
[24]	Hematologic	Targeted therapy	Acadesine	Acadesine	Phase I/II	MTD	DLT	Modified fibonacci	DLT + CR	CR (IWG response criteria)			PK/PD
[25]	Hematologic	Targeted therapy	Venetoclax Ibrutinib	Venetoclax Ibrutinib	Phase I	OBD	DLT + ORR at 2 months	CRM for two binary endpoints		CR (Cheson modified criteria)			
[26]	Solid	Targeted therapy	Angiotensin 1–7	Angiotensin 1–7	Phase I/II	OBD/MTD	DLT + response data for white, platelets and red blood cells	3 + 3					PK
[27]	Solid	Targeted therapy	Emactuzumab	Emactuzumab	Phase I	OBD/MTD	DLT + (PK profile + all response data)	3 + 3		CR (RECIST criteria)		Duration of clinically progression-free follow-up	PK/PD
[28]	Solid	Targeted therapy	Recombinant human thrombopoietin Carboplatin	Recombinant human thrombopoietin	Phase I/II	OBD	Biological response (platelet count response)	Consecutive/sequential cohorts					
[29]	Solid	Targeted therapy	NGR-hTNF Oxaliplatin Capecitabine	NGR-hTNF	Phase I	OBD/MTD	DLT + PK/PD (NGR-hTNF and sTNF receptors 1 and 2)	Consecutive/sequential cohorts		CR (RECIST criteria)		PFS	PK/PD
[30]	Solid	Targeted therapy	Eltrombopag Doxorubicin Ifosfamide	Eltrombopag	Phase I	MTD	DLT	Consecutive/sequential cohorts	Thrombocytopenia + platelet counts				PK/PD
[31]	Solid	Targeted therapy	NGR-hTNF	NGR-hTNF	Phase I	MTD	DLT	Consecutive/sequential cohorts	DLT + CR	CR (RECIST criteria)		PFS, OS	PK/PD
[32]	Solid	Targeted therapy	Cilengitide	Cilengitide	Phase I	OBD	DLT + biological activity rate (BAR)	Consecutive/sequential cohorts		CR (RECIST criteria)			PK/PD
[33]	Solid	Targeted therapy	Celecoxib Erlotinib	Celecoxib	Phase I	OBD	DLT + urinary PGE-M level	Consecutive/sequential cohorts		CR (RECIST criteria)			
[34]	Solid	Targeted therapy	WX-554	WX-554	Phase I	OBD/MTD	DLT + maximal target inhibition	3 + 3		CR (RECIST criteria)			PK/PD
[35]	Solid	Targeted therapy	All-trans-retinoic acid Tamoxifen Alpha-interferon 2a	All-trans-retinoic acid (ATRA)	Phase I	OBD	Biological response	Consecutive/sequential cohorts					PK

*DLT* dose limiting toxicity, IR immune response, CR clinical response, OS overall survival, PFS progression-free survival, TTP time to progression, PK pharmacokinetics, PD pharmacodynamics

^**a**^ Endpoints related to OBD as secondary objective

PK and PD measurements were considered as secondary objectives for the majority of trials (*n* = 12; 54.5%). PK studies included determination of plasma concentration profiles, distribution and clearance of the agent. The clinical response was most often evaluated as per RECIST criteria [37]: 4 (18.1%) for primary endpoint and 16 (72.7%) as secondary endpoint. With regards to survival outcomes, overall and progression free survivals were usually reported (*n* = 9, 40.9%).

Most trials relied on a dose-escalation design based on a single toxicity endpoint (*n* = 20, 90.9%). In such case, DLT was the endpoint used to assess safety of the dose (*n* = 18, 81.8%), otherwise a descriptive analysis of the reported events was provided (n = 2, 9.1%). Most trials (*n* = 19, 86.4%) considered a binary toxicity endpoint defined in terms of the proportion of patients who experienced a dose-limiting toxicity (DLT; yes/no), based on protocol-specific adverse event definitions. Only two articles relied on an adaptive design (Bayesian CRM) which investigated a combination of multiple agents.

## Discussion

We provided an overview of current evidence of phase 1 cancer trials relying on OBD. For those trials specifically reporting the OBD, OBD was considered either as a primary or secondary objective and usually associated with toxicity and efficacy endpoints in order to characterize toxicity with preliminary evidence of efficacy.

The toxicity endpoint was usually defined as a binary variable indicating the presence of DLT during the first cycle of treatment. In the retrieved articles, neither the cumulative toxicity nor DLT beyond the first treatment cycle were considered. Although the definition of efficacy depends on the mechanism of action of the molecule investigated, this review highlights that this definition was heterogeneous and not precisely reported. When the dose-efficacy relationship is non-monotonic, efficacy should be considered. This is particularly relevant for immunotherapy and targeted therapies, where efficacy usually reaches a plateau beyond a given dose. This is typically not the case for cytotoxic agents, for which most designs traditionally assume a monotonic dose-efficacy relationship, since it is expected that increased dose will lead to increased efficacy.

This review also highlights that the term OBD may be misused. Indeed, only two-thirds of manuscripts reporting on OBD actually considered it as a primary objective. For the other third, MTD was the primary objective and dose escalation relied only on the incidence of DLT and did not consider any efficacy data. Such approach may be appropriate to estimate the MTD but will not lead to the assessment of the OBD. In addition, trials targeting OBD as the primary objective should consider both toxicity and efficacy to proceed with dose escalation, which was clearly not the case for most trials as only two proceeded as such.

As a general recommendation, MTD should remain the primary objective in phase 1 trials investigating cytotoxic agents, while efficacy may be assessed as a secondary objective. On the other hand, phase 1 trials for immunotherapeutic and targeted agents should focus on OBD as the primary objective, and should thus jointly account for efficacy and toxicity when proceeding with dose escalation. In this specific setting, two approaches for dose escalation are promising, including designs relying on co-primary endpoints to jointly assess efficacy and toxicity, as well as designs accounting for efficacy only [36]. In this context, different methods exist but they are still underused in practice. These include extensions of the standard CRM in two directions for the modeling of both toxicity and efficacy in a phase I setting. In such extensions, one might consider preserving the bivariate structure of outcomes through a joint modeling of toxicity and efficacy [7]. On the other hand, it is possible to rely on a bivariate distribution for toxicity and efficacy defined either using a binary endpoint (e.g. progression) [38] or a continuous biomarker [8, 9]. More recently, the joint modeling of longitudinal continuous biomarker activity measurements and time to first dose limiting toxicity has also been considered [39]. Finally, incorporating PK, PD or functional imaging as part of dose escalation has also been considered. Up to date, such designs however might be resource intensive which may have limited their application in phase I trials [40]. All these designs rely on the careful collection of all required safety and efficacy parameters, such as clinical and biological parameters.

## Conclusions

OBD should be a primary objective for the assessment of the RP2D as part of targeted therapy or immunotherapy phase I trials in oncology and the statistical methods have to be adapted accordingly.

In the modern era of immunotherapy and targeted treatments, the concept of OBD has become particularly relevant in cancer phase I trials. As such, both toxicity and efficacy should be accounted for in the primary objective of such trials. Phase 1 designs should be adapted accordingly in order to account for both endpoints when proceeding with dose-escalation.

## Supplementary Information

**Additional file 1 Supplementary material 1**. References of the 22 reviewed articles “With OBD”. **Supplementary material 2**. References of the 15 reviewed articles “Without OBD”. **Supplementary material 3**. Prisma Checklist.

## Data Availability

NA

## Abbreviations

AUC: Area under the curve
CRM: Continual reassessment method
DLT: Dose limiting toxicity
CR: Clinical response
IR: Immune response
MTD: Maximum tolerated dose
OBD: Optimal biological dose
OS: Overall survival
PFS: Progression-free survival
PD: Pharmacodynamics
PK: Pharmacokinetics
RP2D: Recommended phase 2 dose
TTP: Time to progression
