# Supplementary material for: Optimal biological dose: a systematic review in cancer phase I clinical trials
Source: BMC Cancer. 2021 Jan 13;21:60. doi: 10.1186/s12885-021-07782-z (PMC7805102; doi:10.1186/s12885-021-07782-z)
Supplement: Supplementary file 1 — Additional file 1 Supplementary material 1. References of the 22 reviewed articles “With OBD”. Supplementary material 2. References of the 15 reviewed articles “Without OBD”. Supplementary material 3. Prisma Checklist. [file 12885_2021_7782_MOESM1_ESM.docx]

**Supplementary Material 1.**

**References of the 22 reviewed articles “with OBD”.**

Cassier PA, Italiano A, Gomez-Roca CA, Le Tourneau C, Toulmonde M, Cannarile MA, et al. CSF1R inhibition with emactuzumab in locally advanced diffuse-type tenosynovial giant cell tumours of the soft tissue: a dose-escalation and dose-expansion phase 1 study. The Lancet Oncology. août 2015;16(8):949‑56.

Cebon J, Jäger E, Shackleton MJ, Gibbs P, Davis ID, Hopkins W, et al. Two phase I studies of low dose recombinant human IL-12 with Melan-A and influenza peptides in subjects with advanced malignant melanoma. Cancer Immun. 16 juill 2003;3:7.

Chawla SP, Staddon A, Hendifar A, Messam CA, Patwardhan R, Kamel YYM. Results of a phase I dose escalation study of eltrombopag in patients with advanced soft tissue sarcoma receiving doxorubicin and ifosfamide. BMC Cancer [Internet]. déc 2013 [cité 9 janv 2020];13(1). Disponible sur: http://bmccancer.biomedcentral.com/articles/10.1186/1471-2407-13-121

Combs SE, Habermehl D, Ganten T, Schmidt J, Edler L, Burkholder I, et al. Phase i study evaluating the treatment of patients with hepatocellular carcinoma (HCC) with carbon ion radiotherapy: The PROMETHEUS-01 trial. BMC Cancer [Internet]. déc 2011 [cité 9 janv 2020];11(1). Disponible sur: http://bmccancer.biomedcentral.com/articles/10.1186/1471-2407-11-67

Geevarghese SK, Geller DA, de Haan HA, Hörer M, Knoll AE, Mescheder A, et al. Phase I/II Study of Oncolytic Herpes Simplex Virus NV1020 in Patients with Extensively Pretreated Refractory Colorectal Cancer Metastatic to the Liver. Human Gene Therapy. sept 2010;21(9):1119‑28.

Gregorc V, Citterio G, Vitali G, Spreafico A, Scifo P, Borri A, et al. Defining the optimal biological dose of NGR-hTNF, a selective vascular targeting agent, in advanced solid tumours. European Journal of Cancer. janv 2010;46(1):198‑206.

Hariharan S, Gustafson D, Holden S, McConkey D, Davis D, Morrow M, et al. Assessment of the biological and pharmacological effects of the ανβ3 and ανβ5 integrin receptor antagonist, cilengitide (EMD 121974), in patients with advanced solid tumors. Annals of Oncology. août 2007;18(8):1400‑7.

Jamieson D, Griffin MJ, Sludden J, Drew Y, Cresti N, Swales K, et al. A phase I pharmacokinetic and pharmacodynamic study of the oral mitogen-activated protein kinase kinase (MEK) inhibitor, WX-554, in patients with advanced solid tumours. European Journal of Cancer. nov 2016;68:1‑10.

Makishima H, Yasuda S, Isozaki Y, Kasuya G, Okada N, Miyazaki M, Mohamad O, Matsufuji N, Yamada S, Tsuji H, Kamada T; Liver Cancer Working Group. [Single fraction carbon ion radiotherapy for colorectal cancer liver metastasis: A dose escalation study.](https://pubmed.ncbi.nlm.nih.gov/30417485/) Cancer Sci. 2019 Jan;110(1):303-309.

Mammoliti S, Andretta V, Bennicelli E, Caprioni F, Comandini D, Fornarini G, et al. Two doses of NGR-hTNF in combination with capecitabine plus oxaliplatin in colorectal cancer patients failing standard therapies. Annals of Oncology. avr 2011;22(4):973‑8.

Miyamoto T, Yamamoto N, Nishimura H, Koto M, Tsujii H, Mizoe J, et al. Carbon ion radiotherapy for stage I non-small cell lung cancer. Radiotherapy and Oncology. févr 2003;66(2):127‑40.

Parihar R. A Phase I Study of Interleukin 12 with Trastuzumab in Patients with Human Epidermal Growth Factor Receptor-2-Overexpressing Malignancies: Analysis of Sustained Interferon Production in a Subset of Patients. Clinical Cancer Research. 1 août 2004;10(15):5027‑37.

Recchia F, De Filippis S, Rosselli M, Saggio G, Cesta A, Fumagalli L, et al. Phase 1B study of subcutaneously administered interleukin 2 in combination with 13-cis retinoic acid as maintenance therapy in advanced cancer. Clin. Cancer Res. mai 2001;7(5):1251‑7.

Reckamp KL. A Phase I Trial to Determine the Optimal Biological Dose of Celecoxib when Combined with Erlotinib in Advanced Non-Small Cell Lung Cancer. Clinical Cancer Research. 1 juin 2006;12(11):3381‑8.

Rodgers KE, Oliver J, diZerega GS. Phase I/II dose escalation study of angiotensin 1-7 [A(1-7)] administered before and after chemotherapy in patients with newly diagnosed breast cancer. Cancer Chemotherapy and Pharmacology. mai 2006;57(5):559‑68.

Toma S, Raffo P, Nicolo G, Canavese G, Margallo E, Vecchio C, et al. Biological activity of all-trans-retinoic acid with and without tamoxifen and alpha-interferon 2a in breast cancer patients. International Journal of Oncology [Internet]. 1 nov 2000 [cité 9 janv 2020]; Disponible sur: http://www.spandidos-publications.com/10.3892/ijo.17.5.991

Vadhan-Raj S, Verschraegen CF, Bueso-Ramos C, Broxmeyer HE, Kudelka AP, Freedman RS, et al. Recombinant Human Thrombopoietin Attenuates Carboplatin-Induced Severe Thrombocytopenia and the Need for Platelet Transfusions in Patients with Gynecologic Cancer. Annals of Internal Medicine. 7 mars 2000;132(5):364.

Van Den Neste E, Cazin B, Janssens A, González-Barca E, Terol MJ, Levy V, et al. Acadesine for patients with relapsed/refractory chronic lymphocytic leukemia (CLL): a multicenter phase I/II study. Cancer Chemotherapy and Pharmacology. mars 2013;71(3):581‑91.

Wages NA, Portell CA, Williams ME, Conaway MR, Petroni GR. Implementation of a Model-Based Design in a Phase Ib Study of Combined Targeted Agents. Clinical Cancer Research. 1 déc 2017;23(23):7158‑64.

Wages NA, Slingluff CL, Petroni GR. A Phase I/II adaptive design to determine the optimal treatment regimen from a set of combination immunotherapies in high-risk melanoma. Contemporary Clinical Trials. mars 2015;41:172‑9.

Wolchok JD, Williams L, Pinto JT, Fleisher M, Krown SE, Hwu W-J, et al. Phase I trial of high dose paracetamol and carmustine in patients with metastatic melanoma: Melanoma Research. avr 2003;13(2):189‑96.

Yau T, Cheng PN, Chan P, Chan W, Chen L, Yuen J, et al. A phase 1 dose-escalating study of pegylated recombinant human arginase 1 (Peg-rhArg1) in patients with advanced hepatocellular carcinoma. Investigational New Drugs. févr 2013;31(1):99‑107.

**Supplementary Material 2.**

**References of the 15 reviewed articles “without OBD”.**

Appels NMGM, Bolijn MJ, Chan K, Stephens TC, Hoctin-Boes G, Middleton M, et al. Phase I pharmacokinetic and pharmacodynamic study of the prenyl transferase inhibitor AZD3409 in patients with advanced cancer. British Journal of Cancer. juin 2008;98(12):1951‑8.

Bahleda R, Le Deley MC, Bernard A, Chaturvedi S, Hanley M, Poterie A, Gazzah A, Varga A, Touat M, Deutsch E, Massard C, Van De Velde H, Hollebecque A, Sallansonnet-Froment M, Ricard D, Taillia H, Angevin E, Ribrag V, Soria JC. [Phase I trial of bortezomib daily dose: safety, pharmacokinetic profile, biological effects and early clinical evaluation in patients with advanced solid tumors.](https://pubmed.ncbi.nlm.nih.gov/29094232/) Invest New Drugs. 2018 Aug;36(4):619-628.

Claringbold PG, Turner JH. NeuroEndocrine Tumor Therapy with Lutetium-177-octreotate and Everolimus (NETTLE): A Phase I Study. Cancer Biotherapy and Radiopharmaceuticals. août 2015;30(6):261‑9.

Combs SE, Kieser M, Habermehl D, Weitz J, Jäger D, Fossati P, et al. Phase I/II trial evaluating carbon ion radiotherapy for the treatment of recurrent rectal cancer: the PANDORA-01 trial. BMC Cancer [Internet]. déc 2012 [cité 9 janv 2020];12(1). Disponible sur: http://bmccancer.biomedcentral.com/articles/10.1186/1471-2407-12-137

Fouliard S, Robert R, Jacquet-Bescond A, du Rieu QC, Balasubramanian S, Loury D, et al. Pharmacokinetic/pharmacodynamic modelling-based optimisation of administration schedule for the histone deacetylase inhibitor abexinostat (S78454/PCI-24781) in phase I. European Journal of Cancer. sept 2013;49(13):2791‑7.

Gallerani E, Zucchetti M, Brunelli D, Marangon E, Noberasco C, Hess D, et al. A first in human phase I study of the proteasome inhibitor CEP-18770 in patients with advanced solid tumours and multiple myeloma. European Journal of Cancer. janv 2013;49(2):290‑6.

Kreitman RJ, Squires DR, Stetler-Stevenson M, Noel P, FitzGerald DJP, Wilson WH, et al. Phase I Trial of Recombinant Immunotoxin RFB4(dsFv)-PE38 (BL22) in Patients With B-Cell Malignancies. Journal of Clinical Oncology. 20 sept 2005;23(27):6719‑29.

van Laarhoven HWM, Fiedler W, Desar IME, van Asten JJA, Marreaud S, Lacombe D, et al. Phase I Clinical and Magnetic Resonance Imaging Study of the Vascular Agent NGR-hTNF in Patients with Advanced Cancers (European Organization for Research and Treatment of Cancer Study 16041). Clinical Cancer Research. 15 févr 2010;16(4):1315‑23.

Manzke O, Tesch H, Borchmann P, Wolf J, Lackner K, Gossmann A, et al. Locoregional treatment of low-grade B-cell lymphoma with CD3×CD19 bispecific antibodies and CD28 costimulation: I. Clinical phase I evaluation. International Journal of Cancer. 15 févr 2001;91(4):508‑15.

Ryan C, Vokes E, Vogelzang N, Janisch L, Kobayashi K, Ratain M. A phase I study of suramin with once- or twice-monthly dosing in patients with advanced cancer. Cancer Chemotherapy and Pharmacology. 1 juill 2002;50(1):1‑5.

[Sessa](https://pubmed.ncbi.nlm.nih.gov/?sort=date&size=200&term=Sessa+C&cauthor_id=12176795) C, [Cuvier](https://pubmed.ncbi.nlm.nih.gov/?sort=date&size=200&term=Cuvier+C&cauthor_id=12176795) C, [Caldiera](https://pubmed.ncbi.nlm.nih.gov/?sort=date&size=200&term=Caldiera+S&cauthor_id=12176795) S, [Bauer](https://pubmed.ncbi.nlm.nih.gov/?sort=date&size=200&term=Bauer+J&cauthor_id=12176795) J, [Van Den Bosch](https://pubmed.ncbi.nlm.nih.gov/?sort=date&size=200&term=Van+Den+Bosch+S&cauthor_id=12176795) S, [Monnerat](https://pubmed.ncbi.nlm.nih.gov/?sort=date&size=200&term=Monnerat+C&cauthor_id=12176795) C, [Semiond](https://pubmed.ncbi.nlm.nih.gov/?sort=date&size=200&term=Semiond+D&cauthor_id=12176795) D, [Pérard](https://pubmed.ncbi.nlm.nih.gov/?sort=date&size=200&term=P%C3%A9rard+D&cauthor_id=12176795) D, [Lebecq](https://pubmed.ncbi.nlm.nih.gov/?sort=date&size=200&term=Lebecq+A&cauthor_id=12176795) A, [Besenval](https://pubmed.ncbi.nlm.nih.gov/?sort=date&size=200&term=Besenval+M&cauthor_id=12176795) M, [Marty](https://pubmed.ncbi.nlm.nih.gov/?sort=date&size=200&term=Marty+M&cauthor_id=12176795) M. Phase I clinical and pharmacokinetic studies of the taxoid derivative RPR 109881A administered as a 1-hour or a 3-hour infusion in patients with advanced solid tumors. Ann Oncol. 2002 Jul;13(7):1140-50.

Simon GR, Garrett CR, Olson SC, Langevin M, Eiseman IA, Mahany JJ, et al. Increased Bioavailability of Intravenous Versus Oral CI-1033, a Pan erbB Tyrosine Kinase Inhibitor: Results of a Phase I Pharmacokinetic Study. Clinical Cancer Research. 1 août 2006;12(15):4645-51.

Steele NL, Plumb JA, Vidal L, Tjørnelund J, Knoblauch P, Buhl-Jensen P, et al. Pharmacokinetic and pharmacodynamic properties of an oral formulation of the histone deacetylase inhibitor Belinostat (PXD101). Cancer Chemotherapy and Pharmacology. juin 2011;67(6):1273-9.

Thistlethwaite FC, Gilham DE, Guest RD, Rothwell DG, Pillai M, Burt DJ, et al. The clinical efficacy of first-generation carcinoembryonic antigen (CEACAM5)-specific CAR T cells is limited by poor persistence and transient pre-conditioning-dependent respiratory toxicity. Cancer Immunology, Immunotherapy. nov 2017;66(11):1425-36.

Wilson PM, El-Khoueiry A, Iqbal S, Fazzone W, LaBonte MJ, Groshen S, et al. A phase I/II trial of vorinostat in combination with 5-fluorouracil in patients with metastatic colorectal cancer who previously failed 5-FU-based chemotherapy. Cancer Chemotherapy and Pharmacology. avr 2010;65(5):979-88.

**Supplementary Material 3.**

**PRISMA CHECKLIST.**

| **Section/topic** | **#** | **Checklist item** | **Reported on page #** |  |
| --- | --- | --- | --- | --- |
| **TITLE** | | |  |  |
| Title | 1 | Identify the report as a systematic review, meta-analysis, or both. | P1 |  |
| **ABSTRACT** | | |  |  |
| Structured summary | 2 | Provide a structured summary including, as applicable: background; objectives; data sources; study eligibility criteria, participants, and interventions; study appraisal and synthesis methods; results; limitations; conclusions and implications of key findings; systematic review registration number. | P2 |  |
| **INTRODUCTION** | | |  |  |
| Rationale | 3 | Describe the rationale for the review in the context of what is already known. | Background, P3, line 61 – P4, line 82 |  |
| Objectives | 4 | Provide an explicit statement of questions being addressed with reference to participants, interventions, comparisons, outcomes, and study design (PICOS). | Background, P4, lines 83 – 91 |  |
| **METHODS** | | |  |  |
| Protocol and registration | 5 | Indicate if a review protocol exists, if and where it can be accessed (e.g., Web address), and, if available, provide registration information including registration number. | N/A |  |
| Eligibility criteria | 6 | Specify study characteristics (e.g., PICOS, length of follow-up) and report characteristics (e.g., years considered, language, publication status) used as criteria for eligibility, giving rationale. | Methods, P4, lines 96 – 101 |  |
| Information sources | 7 | Describe all information sources (e.g., databases with dates of coverage, contact with study authors to identify additional studies) in the search and date last searched. | Methods, P4, lines 96 – 101 |  |
| Search | 8 | Present full electronic search strategy for at least one database, including any limits used, such that it could be repeated. | Methods, P4, lines 96 – 101 |  |
| Study selection | 9 | State the process for selecting studies (i.e., screening, eligibility, included in systematic review, and, if applicable, included in the meta-analysis). | Methods, P4, lines 101 – P5, lines 109 |  |
| Data collection process | 10 | Describe method of data extraction from reports (e.g., piloted forms, independently, in duplicate) and any processes for obtaining and confirming data from investigators. | Methods, P4, lines 101 – P5, lines 109 |  |
| Data items | 11 | List and define all variables for which data were sought (e.g., PICOS, funding sources) and any assumptions and simplifications made. | Methods, P5, lines 113 – 118 |  |
| Risk of bias in individual studies | 12 | Describe methods used for assessing risk of bias of individual studies (including specification of whether this was done at the study or outcome level), and how this information is to be used in any data synthesis. | N/A |  |
| Summary measures | 13 | State the principal summary measures (e.g., risk ratio, difference in means). | Methods, P5, lines 119 – 122 |  |
| Synthesis of results | 14 | Describe the methods of handling data and combining results of studies, if done, including measures of consistency (e.g., I^2^) for each meta-analysis. | N/A |  |
| **Section/topic** | **#** | **Checklist item** | **Reported on page #** | |
| Risk of bias across studies | 15 | Specify any assessment of risk of bias that may affect the cumulative evidence (e.g., publication bias, selective reporting within studies). | N/A | |
| Additional analyses | 16 | Describe methods of additional analyses (e.g., sensitivity or subgroup analyses, meta-regression), if done, indicating which were pre-specified. | N/A | |
| **RESULTS** | | |  | |
| Study selection | 17 | Give numbers of studies screened, assessed for eligibility, and included in the review, with reasons for exclusions at each stage, ideally with a flow diagram. | Results, P5, lines 126 – 133  P13, Figure 1 | |
| Study characteristics | 18 | For each study, present characteristics for which data were extracted (e.g., study size, PICOS, follow-up period) and provide the citations. | Results, P5, line 135 – P6, line 149  P14, Table 1  Supplementary Material 1.  Supplementary Material 2. | |
| Risk of bias within studies | 19 | Present data on risk of bias of each study and, if available, any outcome level assessment (see item 12). | N/A | |
| Results of individual studies | 20 | For all outcomes considered (benefits or harms), present, for each study: (a) simple summary data for each intervention group (b) effect estimates and confidence intervals, ideally with a forest plot. | P15, Table 2 | |
| Synthesis of results | 21 | Present results of each meta-analysis done, including confidence intervals and measures of consistency. | N/A | |
| Risk of bias across studies | 22 | Present results of any assessment of risk of bias across studies (see Item 15). | N/A | |
| Additional analysis | 23 | Give results of additional analyses, if done (e.g., sensitivity or subgroup analyses, meta-regression [see Item 16]). | N/A | |
| **DISCUSSION** | | |  | |
| Summary of evidence | 24 | Summarize the main findings including the strength of evidence for each main outcome; consider their relevance to key groups (e.g., healthcare providers, users, and policy makers). | Discussion, P7, lines 177 – P8, line 215 | |
| Limitations | 25 | Discuss limitations at study and outcome level (e.g., risk of bias), and at review-level (e.g., incomplete retrieval of identified research, reporting bias). | N/A | |
| Conclusions | 26 | Provide a general interpretation of the results in the context of other evidence, and implications for future research. | Conclusions, P8, lines 217 – 224 | |
| **FUNDING** | | |  | |
| Funding | 27 | Describe sources of funding for the systematic review and other support (e.g., supply of data); role of funders for the systematic review. | P9, lines 243 – 246 | |
